# 3DPointCaps++: Learning 3D Representations with Capsule Networks

**DOI:** 10.1007/s11263-022-01632-6

**Published:** 2022-07-30

**Authors:** Yongheng Zhao, Guangchi Fang, Yulan Guo, Leonidas Guibas, Federico Tombari, Tolga Birdal

**Affiliations:** 1 Informatics at Technische Universität München, Munich, Germany; 2 School of Electronics and Communication Engineering, The Shenzhen Campus of Sun Yat-sen University, Shenzhen, China; 3 Computer Science Department, Stanford University, Stanford, CA USA; 4 Google, Mountain View, CA USA; 5 Department of Computing, Imperial College London, London, UK

**Keywords:** Capsule networks, 3D Point clouds, Representation learning, Autoencoder, Unsupervised learning, 3D Reconstruction, 3D Shapes

## Abstract

We present **3DPointCaps++** for learning robust, flexible and generalizable 3D object representations without requiring heavy annotation efforts or supervision. Unlike conventional 3D generative models, our algorithm aims for building a structured latent space where certain factors of shape variations, such as object parts, can be disentangled into independent sub-spaces. Our novel decoder then acts on these individual latent sub-spaces (i.e. capsules) using deconvolution operators to reconstruct 3D points in a self-supervised manner. We further introduce a cluster loss ensuring that the points reconstructed by a single capsule remain local and do not spread across the object uncontrollably. These contributions allow our network to tackle the challenging tasks of part segmentation, part interpolation/replacement as well as correspondence estimation across rigid / non-rigid shape, and across / within category. Our extensive evaluations on ShapeNet objects and human scans demonstrate that our network can learn generic representations that are robust and useful in many applications.

## Introduction

Differently from appearance-based cues as the ones provided by images, 3D data supplies information concerning the structure and shape of the physical environment, thus widening our capabilities for modeling the world around us. The richness of the information provided by 3D cues allows us to reason both at a high, abstract level (e.g. in terms of semantics, affordances, functions) as well as at a low level, closer to data (e.g. geometry, flow, reflectance). For this reason, recent years have witnessed a significant leap forward in the amount of 3D data acquired, processed and generated especially in the fields of robotics, autonomous driving and computer graphics applications (Sun et al. 2019; Caesar et al. 2019). However, the extent at which we can harness the power of 3D depends largely upon the specific data-driven *representations* that we are able to learn through the algorithms which are tasked with the analysis of 3D data. A good 3D data representation is interpretable and can disentangle the underlying data generation mechanisms while paving the way for several relevant applications, such as reconstruction, classification, generative modeling among many others (Rempe et al. 2020; Quessard et al. 2020). In the 3D domain, an additional challenge towards accomplishment of these tasks is posed by the non-standard, often non-regular structure of the input data, as well as the complexity of transformations that the scene undergoes, i.e. the curse of dimensionality ’causes an exponential growth in the number of parameters used to represent transformations.

**Fig. 1:**
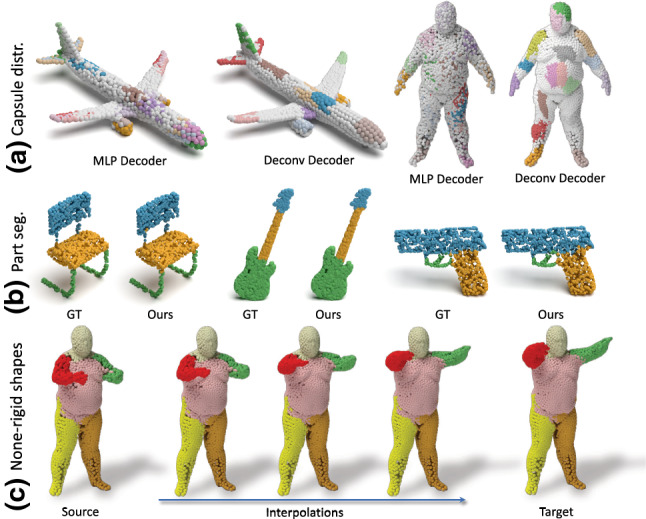
3DPointCaps++ can learn representations of 3D objects that support multiple desirable properties such as a disentangled latent space and local spatial attention (**a**). These properties enable different interesting applications such as part segmentation (**b**) or non-rigid part interpolation/replacement (**c**)

To address this challenge, we extend upon our earlier work on 3D point capsule networks (3DCapsNets) Zhao et al. (2019) and propose *3DCapsNets++*. Our pursuit is to learn an object-specific structured latent representation decomposed into several subspaces, each of which is responsible for a degree of freedom that, when changed, independently and tractably, modifies the generated 3D data. We consume the 3D input in the form of a point cloud to introduce flexibility in the input modality i.e. point sets can explain a variety of modalities such as depth maps, CAD-meshes, fusion models or laser scans. To natively operate on the point clouds we utilize the prevalent PointNets Qi et al. (2017a), whereas to create a rich latent space we make use of the popular capsule networks. To facilitate unsupervised learning of these representations, we propose a deep encoder-decoder architecture, where the encoder involves generating an intermediary over-parameterized set of latent features, *primary capsules*, using several PointNets initialized randomly. We employ a *dynamic routing* Sabour et al. (2017) algorithm to cluster these capsules into their latent counterparts. To ensure that each latent subspace instantiates a particular object property, we propose novel *Deconvolutional* (Deconv) layers in addition to the multi-layer perceptrons (MLPs) employed in previous work such as AtlasNet Groueix et al. (2018a) or 3DCapsNets Zhao et al. (2019). We show that these Deconv layers, when trained with our novel *unsupervised clustering loss*, not only yield better reconstruction accuracy, but also distribute themselves in a conglomerated fashion across the object as shown in Fig. 1a.

Our extensive experimental evaluation demonstrates that, in the quest of learning expressive, distinctive and flexible representations, our 3D capsule network takes important steps: (i) We are able to achieve state-of-the-art reconstruction accuracy both on rigid and non-rigid shapes using a fraction of the training data; (ii) The structure of the latent space enables novel applications, such as 3D part interpolation (Fig. 1c) and replacement on both rigid and non-rigid shapes without data specific customizations, i.e. our architecture remains identical for both kinds of input; (iii) Thanks to the local spatial attention of each capsule, our model allows for part segmentation (Fig. 1) using a very limited amount of training data.

Disembarking from our previous work Zhao et al. (2019), we set forth the following contributions:

- We present a new Deconvolutional capsule decoder that shows better specialization properties compared to the state of the art.
- We introduce a new initialization procedure through a cluster loss to ensure the desired diversity and clustering in the generative direction.
- We show, for the first time, how this architecture can learn non-rigid representations besides the rigid ones, and demonstrate this ability on the human body models.

## Related Work

*Point clouds in deep networks.* Thanks to their apability of efficiently explaining 3D data without making assumptions on the modality, point clouds are the preferred containers for many 3D applications (Zhou and Tuzel 2018; Naseer et al. 2019). Due to this widespread use, recent works such as PointNet Qi et al. (2017a), PointNet++ Qi et al. (2017b), SO-Net Li et al. (2018), spherical convolutions Lei et al. (2018), Monte Carlo convolutions Hermosilla et al. (2018) dynamic graph networks Wang et al. (2019), KPConv Thomas et al. (2019), MinkowskiNet Gojcic et al. (2021); Choy et al. (2019) or relation-shape (RS) CNN Liu et al. (2019) have all devised point cloud-specific architectures that exploited the sparsity and permutation-invariant properties of 3D point sets. A parallel track of works processed point sets by using local projections reducing the convolution operation down to two dimensions (Tatarchenko et al. 2018; Huang et al. 2018). Additionally, there is a large body of works that exploit locality in a more explicit way, such as PPFNet Deng et al. (2018b), PPF-FoldNet (Deng et al. 2018a, 2019), FCGF (Choy et al. 2019; Gojcic et al. 2020). Similar to our work, recent methods extended the point cloud networks that operate on rigid shapes or 3D scenes to the case of deformable bodies (Poulenard and Ovsjanikov 2018; Uy et al. 2020).

Recently, unsupervised architectures followed up on their supervised counterparts. PU-Net Yu et al. (2018) proposed better upsampling schemes to be used in decoding. FoldingNet Yang et al. (2018) introduced the idea of deforming a 2D grid to decode a 3D surface as a point set. PPF-FoldNet Deng et al. (2018a) improved upon the supervised PPFNet Deng et al. (2018b) in local feature extraction by benefiting from FoldingNet’s decoder Yang et al. (2018). AtlasNet Groueix et al. (2018a) can be seen as an extension of FoldingNet to multiple grid patches and provided extended capabilities in data representation. 3D-Coded Deprelle et al. (2019) has also proposed to learn those patches called *templates*. PointGrow Sun et al. (2018) devised an auto-regressive model for both unconditional and conditional point cloud generation leading to effective unsupervised feature learning. Achlioptas et al. (2018) adapted GANs to 3D point sets, paving the way to enhanced generative learning. Both CaSPR Rempe et al. (2020) and PointFlow Yang et al. (2019) used normalizing flows for learning invertible representations. Yet, it is known that such strict invertibility might harm universal approximation capabilities Kong and Chaudhuri (2020).

*2D capsule networks.* Thanks to their general applicability, capsule networks (CNs) have found tremendous use in 2D deep learning. LaLonde and Bagci LaLonde and Bagci (2018) developed a deconvolutional capsule network, called *SegCaps*, tackling object segmentation. Duarte et al. (2018) extended CNs to action segmentation and classification by introducing *capsule-pooling*. Jaiswal et al. (2018), Saqur and Vivona (2018) and Upadhyay and Schrater (2018) proposed Capsule-GANs, i.e. capsule network variants of the standard generative adversarial networks (GAN) Goodfellow et al. (2014). These have shown better 2D image generation performance. Lin et al. (2018) showed that capsule representations learn more meaningful 2D manifold embeddings than neurons in a standard CNN do.

**Fig. 2:**
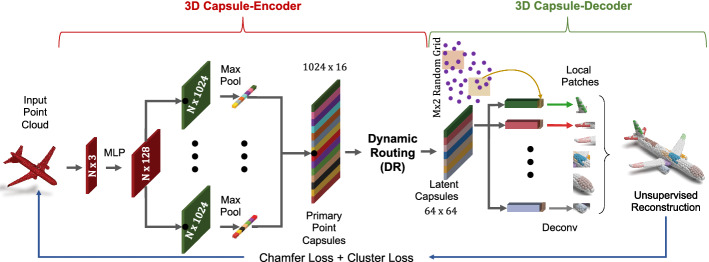
Proposed 3DPointCaps++ architecture based on the 3D Capsule-encoder and Deconvolution decoder

Kosiorek et al. proposed an unsupervised capsule autoencoder (SCAE), which explicitly uses geometric relationships between parts to reason about objects in a viewpoint independent fashion Kosiorek et al. (2019). Sabour et al.exploited the fact that regions of the image that move together often belong together and introduced flow capsules Sabour et al. (2020). Ramirez et al.devised Bayesian capsule networks for estimating 3D human poses from 2D images Ramirez et al. (2020). Afshar et al.proposed Covid-Caps Afshar et al. (2020) for identifying positive COVID-19 cases based on X-Ray images.

After the original dynamic routing (DR) Sabour et al. (2017) has been widely adopted, scholars questioned whether this DR is the optimal routing strategy. This motivated several works dedicated to improve upon the initial CN proposal. Hinton et al.improved the routing by the EM algorithm  Hinton et al. (2018). Wang and Liu considered the routing as an optimization minimizing a combination of clustering-like loss and a KL regularization term Wang and Liu (2018). Chen and Crandall Chen and Crandall (2018) suggested *trainable routing* for better clustering of capsules. Zhang et al. (2018) unified the existing routing methods under one umbrella and proposed weighted kernel density estimation based routing methods. Zhang et al. (2018) chose to use the norm to explain the existence of an entity and proposed to learn a group of capsule subspaces onto which an input feature vector is projected. Lenssen et al. (2018) introduced guaranteed equivariance and invariance properties to capsule networks by the use of group convolutions. Ribeiro et al.successfully identified the drawbacks of current capsule networks and proposed a globally aware routing algorithm that represents the inherent uncertainty in part-object relationships Ribeiro et al. (2020). A recent study which analyzes different routing methods, concludes that capsule networks need an improved routing algorithm Paik et al. (2019). Nevertheless, in our work we stick with the original dynamic routing as proposed by Sabour et al. (2017).

*3D capsule networks.* Following the advances in 2D domain, numerous 3D vision algorithms employed capsule networks to achieve either more accurate or more capable methods. Weiler et al. (2018) rigorously formalized the convolutional capsules and presented a convolutional neural network (CNN) equivariant to rigid motions. Jiménez-Sánchez et al. (2018) as well as Mobniy and Mobiny and Van Nguyen (2018) extended capsules to deal with volumetric medical data. VideoCapsuleNet Duarte et al. (2018) also used a volumetric representation to handle temporal frames of the video. Our 3DPointCapsNets Zhao et al. (2019) was the first to extend the capsule networks to consider 3D point clouds in their raw form inspiring a new frontier in capsule networks. Point2SpatialCapsule Wen et al. (2020) further improved the ability to learn local spatial relationships. Geometric capsule networks Srivastava et al. (2019) used a multi-view agreement voting mechanism to discover an object’s canonical pose and its pose-invariant feature vector. Zhao et al. (2020) proposed quaternion equivariant capsule modules for making the capsule networks truly equivariant to the *SO*(3) rotations of point clouds, thanks to the use of local reference frames. Similar to CaSPR Rempe et al. (2020), Sun et al. (2020) learned canonical representations for 3D shapes via capsule networks.

## Method

We now explain the specifics of our method. We start with defining formally the terms used in the rest of the paper while providing the theoretical motivation and background (§ 3.1). We then proceed to the specifics of our architecture (*c.f.* Fig. 2) delving into the *encoder* and *decoder*, subsequently in § 3.2. We finally explain the unsupervised and (optionally) supervised cues used to train our network.

### Formulation

We first follow the AtlasNet convention Groueix et al. (2018a) and present a unified view of some of the common 3D auto-encoders. Then, we explain both our MLP and Deconv based *3d point capsule networks* within this geometric perspective and justify their superiority compared to their predecessors. We will start by recalling the basic concepts:

#### Definition 1

(Surface and Point Cloud) A 3D surface (*shape*) is a differentiable 2-manifold embedded in the ambient 3D Euclidean space: $\mathcal {M}^2\in \mathbb {R}^3$. We approximate a **point cloud** as a sampled discrete subset of the surface $\mathbf {X}=\{\mathbf {x}_i \in \mathcal {M}^2 \cap \mathbb {R}^3\}$.

#### Definition 2

(Diffeomorphism) A diffeomorphism is a continuous, invertible, structure-preserving map between two differentiable surfaces.

#### Definition 3

(Chart and Parametrization) We admit an open set $U\in \mathbb {R}^2$ and a diffeomorphism $C: \mathcal {M}^2 \mapsto U\in \mathbb {R}^2$ mapping an open neighborhood in 3D to its 2D local coordinates. *C* is called a **chart**. Its inverse, $\Psi \equiv C^{-1}: \mathbb {R}^2\mapsto \mathcal {M}^2$ is called a **parameterization**.

#### Definition 4

(Atlas) A set of charts with images covering the 2-manifold is called an **atlas**: $\mathcal {A}=\cup _i C_i(\mathbf {x}_i)$.

A 3D auto-encoder learns to generate a 3D surface $\mathbf {X}\in \mathcal {M}^2 \cap \mathbb {R}^{N \times 3}$. By virtue of Definition 3$\Psi$ deforms a 2D point set to a surface. The goal of the generative models that are of interest here is to learn $\Psi$ to best reconstruct $\hat{\mathbf {X}}\approx \mathbf {X}$:

#### Definition 5

(Problem) Learning to generate the 2-manifolds is defined as finding function(s) $\Psi (U\,|\,{\varvec{\theta }})\,:\, \Psi (U\,|\,{\varvec{\theta }})\approx \mathbf {X}$ Groueix et al. (2018a). ${\varvec{\theta }}$ is a lower dimensional parameterization of these functions: $|{\varvec{\theta }}|<|\mathbf {X}|$.

#### Theorem 1

Given that $C^{-1}$ exists, $\Psi$, chosen to be a 3-layer MLP, can reconstruct arbitrary 3D surfaces.

#### Proof

The proof is given in Yang et al. (2018) and follows from the universal approximation theorem (UAT).

#### Theorem 2

There exists an integer *K* s.t. an MLP with *K* hidden units universally reconstruct $\mathbf {X}$ up to a precision $\epsilon$.

#### Proof

The proof follows trivially from Theorem 1 and UAT Groueix et al. (2018a).

Given these definitions, some of the typical 3D point decoders differentiate by making four choices (Qi et al. 2017a; Groueix et al. 2018a; Yang et al. 2018):

1. An open set *U* or discrete grid ${\mathbf {U}}\equiv \mathbf {P}=\{\mathbf {p}_i \in \mathbb {R}^2\}$.
2. Distance function $d(\mathbf {X},\hat{\mathbf {X}})$ between the reconstruction $\hat{\mathbf {X}}$ and the input shape $\mathbf {X}$.
3. Parameterization function(s) $$\Psi $$.
4. Parameters $$({\varvec{\theta }})$$ of $$\Psi $$: $$\Psi (U\,|\, {\varvec{\theta }})$$.

**Fig. 3:**
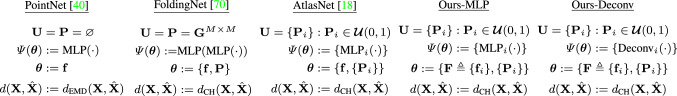
Well known 3D auto-encoders vs. the proposed formulations

**Fig. 4:**
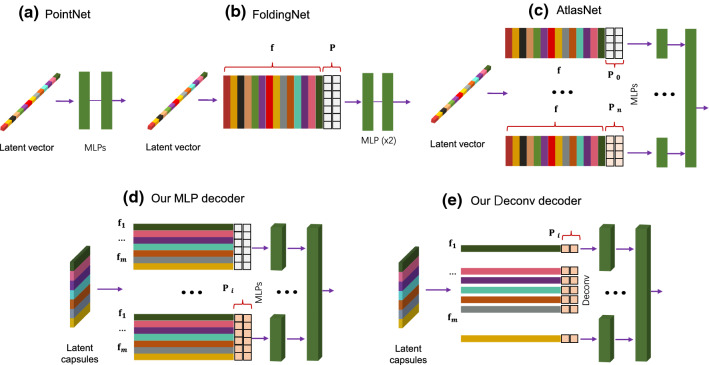
Comparison of four different state-of-the-art 3D point decoders. PointNet uses a single latent vector, and no surface assumption. Thus, ${\varvec{\theta }}_{\text {pointnet}}=\mathbf {f}$. FoldingNet Yang et al. (2018) learns a 1D latent vector along with a fixed 2D grid ${\varvec{\theta }}_{\text {folding}}=\{ \mathbf {f},\,\mathbf {P}\}$. The advanced AtlasNet Groueix et al. (2018a) learns to deform multiple 2D configurations onto local 2-manifolds: ${\varvec{\theta }}_{\text {atlas}}=\{ \mathbf {f},\,\{\mathbf {P}_i\}\}$. Our point-capsule-network is capable of learning multiple latent representations each of which can fold a distinct 2D grid onto a specific local patch, ${\varvec{\theta }}_{\text {ours}}=\{ \{\mathbf {f}_i\},\,\{\mathbf {P}_i\}\}$. Note that both of the MLP and Deconv variants have similar latent representations with different parameterization functions, i.e. the way the grid points are deformed

One of the first works in this field, *PointNet* Qi et al. (2017a) is extended naturally to an AE by Achlioptas et al. (2018) making arguably the simplest choice. It lacks the grid structure $U=\varnothing$ and functions $\Psi$ only depend upon a single latent feature: $\Psi (U\,|\,{\varvec{\theta }})=\Psi ({\varvec{\theta }})=\text {MLP}(\cdot \,|\,\mathbf {f}\in \mathbb {R}^k)$. FoldingNet uses a two-stage MLP as $\Psi$ to warp a fixed grid $\mathbf {P}$ onto $\mathbf {X}$. A transition from FoldingNet to AtlasNet requires having multiple MLP networks operating on multiple 2D sets $\{\mathbf {P}_i\}$ constructed randomly on the domain $]0,1[^2$: ${\varvec{\mathcal {U}}}(0,1)$. These explain the improved learning capacity of AtlasNet: different MLPs learn to reconstruct distinct local surface patches by learning different charts.

Unfortunately, while numerous charts can be defined in the case of AtlasNet, all the methods above still rely on a single latent feature vector, replicated and concatenated with *U* to create the input to the decoders. However, point clouds are found to consist of multiple basis functions Sung et al. (2018) and having a single representation governing them all is not optimal. We opt to go beyond this restriction and choose to have a set of latent features $\{\mathbf {f}_i\}$ to capture different, meaningful basis functions. With the aforementioned observations we can now re-write the well known 3D auto-encoders and introduce two decoder formulations (*MLP* and *Deconv*) as shown in Fig. 3. Note the difference between these two variants lies in the choice of parameterizations and will be made clearer when discussing the specifics of the architectures (§ 3.2). In the figure, $d_\text {{EMD}}$ refers to the Earth Mover distance Rubner et al. (2000) and $d_\text {{CH}}$ is the Chamfer distance. $\mathbf {G}^{M\times M}=\{(i \otimes j) : \forall i,j \in [0,\dots ,\frac{M-1}{M}]\}$ depicts a 2D uniform grid. $\mathbf {f}\in \mathbb {R}^{k}$ represents a *k*-dimensional latent vector. ${\varvec{\mathcal {U}}}(a,b)$ denotes an open set defined by a uniform random distribution in the interval $]a,b[^2$.

Note that it is possible to easily mix these choices to create variations^1^. Though, many interesting architectures only optimize for a single latent feature $\mathbf {f}$. To the best of our knowledge, one promising direction is taken by the capsule networks Hinton et al. (2011), where multitudes of convolutional filters enable the learning of a collection of *capsules* $\{\mathbf {f}_i\}$ thanks to the dynamic routing Sabour et al. (2017). Hence, our earlier work Zhao et al. (2019) and this paper combined, we learn the parameters $\{{\varvec{\theta }}_i\}$ by devising a new point cloud *capsule decoder* that we coin *3D-PointCapsNet*. In addition to Zhao et al. (2019) we also present the *Deconv* version with improved parameterizations. We further illustrate the choices made by four AEs under this unifying umbrella in Fig. 4.

Rationale for Different Parameterization Functions: Unsupervised autoencoders for shape generation suffer from the lack of control in the distribution, smoothness or overlap of the reconstructed points. In fact, Groueix et al. (2018a) explain the spatial attention of the patches by the laziness of the neural network, as a specific mechanism to regularize the reconstructions is missing. However, an explicit control either in the form of spatial spread or patch stitching is helpful in multiple fronts such as correspondence estimation or part manipulation. In our view of the problem, such capability can be injected into the parameterization functions $\Psi$ such that the reconstructions follow certain desirable properties. To this end, besides the MLP based methods like AtlasNet Groueix et al. (2018a) or 3DPointCapsNets Zhao et al. (2019), we also introduce deconvolutional decoders. In the sequel, we will explain the specifics of these contributions.

### 3D-PointCapsNet Architecture

Our descriptions here will follow Fig. 2 where our pipeline is illustrated.

#### Encoder

The Input to our network is an $N \times d$ point cloud, where we fix $N=2048$ for 3D object and $N=4096$ for 3D human body, and for typical point sets $d=3$. Similarly to PointNet Qi et al. (2017a), we use a point-wise Multi-Layer Perceptron (MLP) $(3-64-128-1024)$ to extract individual local feature maps. In order to diversify the learning as suggested by capsule networks (Sabour et al. 2017; Hinton et al. 2011), we feed these feature maps into multiple independent convolutional layers with different weights, each with a distinct summary of the input shape with diversified attention. We then max-pool their responses to obtain a global latent representation. These descriptors are then concatenated into a set of vectors termed *primary point capsules* (PPC), $\mathbf {F}$. Size of $\mathbf {F}$ depends upon the size $S_c:=1024$ and the number $K:=16$ of independent kernels at the last layer of MLP. While many other mechanisms can be used for generating PPC, we found this simple procedure to work well also for 3D point networks. We then use the *dynamic routing* Sabour et al. (2017) to embed the primary point capsules into higher level *latent capsules*. Each capsule is independent and can be considered as a *cluster centroid* (codeword) of the primary point capsules. The total size of the latent capsules is fixed to $64\times 64$ (i.e. , 64 vectors each sized 64). Essentially, dynamic routing acts like a soft-kmeans clustering algorithm (Ren and Lu 2018; Malmgren 2019) simultaneously determining the centroids and the assigments from primary to the latent capsules.

#### Decoder

To endow our network with the reconstruction capability, we propose two decoder architectures based upon: (i) multi-layer perceptron (MLP) and (ii) Deconvolution (Deconv).

MLP Decoder: Our MLP-based decoder treats the latent capsules as a feature map and uses an MLP$(64-64-32-16-3)$ to reconstruct a patch of points $\hat{\mathbf {X}}_i$, where $|\hat{\mathbf {X}}_i|=64$. Instead of replicating a single latent vector as in (Yang et al. 2018; Groueix et al. 2018a), we replicate the entire capsule *m* times and to each replica we append a unique randomly synthesized grid $\mathbf {P}_i$ specializing it to a region on the output shape. This further stimulates the diversity. We arrive at the final shape $\hat{\mathbf {X}}_i$ by propagating the replicas through a final MLP for each patch and gluing the output patches together. We choose $m =32$ (the number of patches) to reconstruct $|\hat{\mathbf {X}}|=32\times 64=2048$ points for the rigid objects and $m =64$ to reconstruct $|\hat{\mathbf {X}}|=64\times 64=4096$ points for the non-rigid shapes. Note that the number of points reconstructed is always the same as the cardinality of the input input.

Lack of Local Attention in MLP-Decoder: Unfortunately, as demonstrated in (Zhao et al. 2019) and as we will further show in § 4, such an MLP decoder cannot ensure that individual capsules reconstruct particular patches of the shape, unless supervised. We now explain the reason behind this. During learning, the dynamic routing algorithm can learn geometric information from the training data, and thus guides the capsules to focus on certain parts. Meanwhile, the correspondences between capsules and reconstructed points are weak and highly dependent upon the global object shape and the dataset. For instance, some capsules can be split into two parts. This is mainly due to the symmetry property of CAD models in the ShapeNet dataset. Besides, when the size and the training data is not rich in terms of geometric complexity, it is difficult for capsules to concentrate on local areas and hence on certain object parts (Fig. 1a). Consequently, points tend to spread over the reconstructed shape.

To tackle this problem, we: (i) introduce a Deconvolution (Deconv) structure into the decoder and (ii) develop an auxiliary cluster loss function to improve the local spatial attention capability of the 3D point capsule networks.

Deconvolutional Decoder (Deconv): Our Deconv decoder takes one latent capsule as its input and similar to Fan et al. (2017) uses a deconvolutional layer, Deconv$(64\times 1-32\times 2-16\times 4-4\times 16-3\times |\hat{\mathbf {X}}_i|)$ to generate a set of points. Deconvolution is used to upsample latent capsules as well as for feature extraction. For the former, we concatenate a single grid point to a capsule and deconvolve it to yield $|\hat{\mathbf {X}}_i|=32$ points for the rigid objects and $|\hat{\mathbf {X}}_i|=64$ for the human body. This operation changes the feature channels from 64 to 3 (spatial location of reconstructed points). This way, the upsampling stage gathers the nearby feature vectors resulting in continuous and smooth maps unlike our MLP parameterization. This directly links the latent capsules to the reconstructed patches. A neighborhood around a single grid point in the 2D local coordinates should be lifted to a region in 3D. As we will empirically demonstrate later in § 4, such design will yield inherently smooth reconstructions since input feature vectors are overlapped and summed up during the Deconv operation. This is also confirmed by Fan et al. (2017) and attributed to the spatial continuity of the deconvolution.

Note that our Deconv based decoder is not a drop-in replacement for the MLP-decoder across all applications. The latent feature learnt from our deconvolutional architecture focuses on local parts of an input point cloud. This may be helpful in learning local information, resulting in the improvement of the capsule classification (part segmentation). However, it may not benefit the learning of a global object context. Hence, these different decoders have different performances across different tasks, i.e. features learned from MLP-based decoder show higher discriminative power and performs better on classification task whereas Deconv has better performance e.g. in correspondence estimation and part segmentation.

#### Training Losses

Similar to other autoencoders, we train our deep network using a distance metric on the original and the reconstructed point clouds. In addition, we also regularize the training to allow for a control over the distribution of the reconstructed points. Optionally, we could incorporate task dependent supervision cues depending upon their availability.

Chamfer Loss Our autoencoder is guided in a self supervised manner by the discrete Chamfer metric, which approximates the similarity of 2-manifolds:

1 $$\begin{aligned}&\mathcal {L}_{CH}(\mathbf {X},\hat{\mathbf {X}})\nonumber \\&\quad =\frac{1}{|\mathbf {X}|}\sum \limits _{\mathbf {x}\in \mathbf {X}} \min _{\hat{\mathbf {x}}\in \hat{\mathbf {X}}} \Vert \mathbf {x}-\hat{\mathbf {x}} \Vert _2 + \frac{1}{|\hat{\mathbf {X}}|}\sum \limits _{\hat{\mathbf {x}} \in \hat{\mathbf {X}}} \min _{\mathbf {x}\in \mathbf {X}} \Vert \mathbf {x}-\hat{\mathbf {x}} \Vert _2 \end{aligned}$$

where $\hat{\mathbf {X}}$ follows from the parameterization acting on capsules: $\hat{\mathbf {X}} = \cup _i \Psi _i(\mathbf {P}_i | \{\mathbf {f}_i\})$.

Cluster Loss For 3D shapes, *locality* is a desirable property. For instance, CaSPR Rempe et al. (2020) demonstrated that representations learned without locality, i.e. using PointNet Qi et al. (2017a) instead of PointNet++ Qi et al. (2017b), cannot support pose canonicalization even under heavy supervision. Hence, to enhance the learned representations and to allow for applications such as part segmentation or part replacement (see § 4.3), we seek to encourage certain locally specialized spatial attention. Unfortunately, neither AtlasNet Groueix et al. (2018a) nor 3DPointCapsNets Zhao et al. (2019) have an explicit mechanism to this end. Both works rely upon locality being an emergent property rather than a built-in one. To go beyond that, we softly impose a *clustering* loss so as to concentrate the points reconstructed by a single capsule on a single local region, making those points as close and grouped as possible. With that aim, we develop a *cluster loss* and constrain the point reconstruction of each capsule. In other words, such cluster loss minimizes the inter-point distance between per each point set reconstructed from a single capsule:

2 $$\begin{aligned} \mathcal {L}_{cluster}(\mathbf {C})=\frac{1}{N}\sum \limits _{\mathbf {C}_{n} \in \mathbf {C}}\max _{\mathbf {x}_{i}, \mathbf {x}_{j} \in \mathbf {C}_{n}} \Vert \mathbf {x}_{i}-\mathbf {x}_{j} \Vert _2 \end{aligned}$$

Here $\mathbf {C}_{n}$ and $\mathbf {C}$ denote the reconstruction obtained from the $n^{th}$ latent capsule and from all the capsules, respectively. By minimizing this term, we minimize the squared distance to the farthest point in the local patch, similar to that of PointCleanNet Rakotosaona et al. (2020).

Note that such a loss would degenerate immediately to a single point if not used in conjunction with a *data term*. Hence, our final training loss is a combination of reconstruction and cluster losses weighted by a balancing scalar $\lambda$:

3 $$\begin{aligned} \mathcal {L}(\mathbf {X},\hat{\mathbf {X}},C) = \mathcal {L}_{CH}(\mathbf {X},\hat{\mathbf {X}}) + \alpha \mathcal {L}_{cluster}(\mathbf {C}) \end{aligned}$$

Note that the constraint imposed by the cluster loss will effect the convergence and thus the reconstruction accuracy, unless relaxed. To ensure that both points are reconstructed accurately and local spatial attention is attained, we follow an adaptive strategy in choosing $\lambda$. In the first 10-epochs, we set it to a relatively high value $\lambda =0.1$ and gradually decrease in the following epochs.

**Fig. 5:**
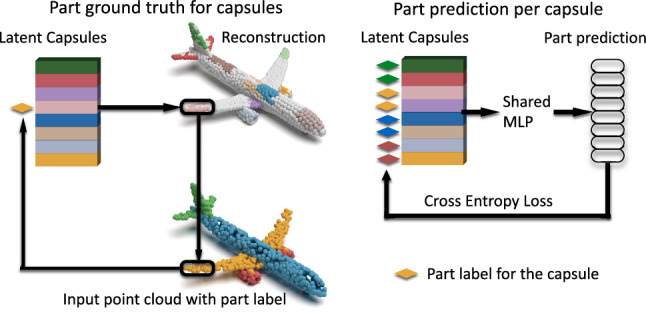
Capsule-Part Association. Supervising the 3d point capsule networks for part prediction. Instead of performing a point-wise part labeling, we use a capsule-wise association requiring less data annotation efforts

Incorporating Optional Supervision We now demonstrate how additional supervision signals can be used within the context of capsules. While the aforementioned losses can attain local attention without supervision, learning semantically meaningful information such as rigid or non-rigid part segmentation might still need supervision. Thus, we use capsule-level part associations as particular cues for showing how add additional, optional supervision structure into our network.

Motivated by the regularity of capsule distribution over the 2-manifold, we propose a capsule-part network that spatially segments the object by associating capsules to parts. The goal here is to assign each capsule to a single part of the object. Hence, we treat this part-segmentation task as a per-capsule classification problem, rather than a per-point one as done in various preceding algorithms (Qi et al. 2017a, b). This is only possible due to the spatial attention of the capsule networks. Note, our algorithm can theoretically use a single capsule to represent each part. In practice, this can lead to two problems: (i) the boundary of capsules may be inconsistent with the ground truth due to the reduction of annotation and the flexibility to represent the transitions; (ii) more importantly, capsules with a constant number of points is hard to represent parts at various scales (e.g hand and leg). For these reasons, we choose to use different number of capsules to represent one part. Investigating with dynamically assigned number of points during inference is an interesting research direction and we leave it for a future study.

The input of our capsule-part network are the latent-capsules obtained from the pre-trained encoder. The output is the part label for each capsule. The ground truth (GT) capsule labeling is obtained from the ShapeNet-Part dataset Yi et al. (2016) in three steps: 1) reconstructing the local part given the capsule and a pre-trained decoder, 2) retrieving the label of the nearest neighbor (NN) GT point for each reconstructed point, 3) computing the most frequent one (mode) among the retrieved labels.

To associate a part to a capsule, we use a shared MLP with a cross entropy loss classifying the latent capsules into parts. This network is trained independently from the 3D-PointCapsNet AE for part supervision.

The pipeline of our propose part segmentation algorithm is demonstrated in Fig. 5. First, we assign the GT part label of each point (e.g. right wing of the airplane on the bottom) to the corresponding capsule. The label of the capsule is determined as the mode of the labels assigned to it. The reconstruction with spatial attention (airplane on the top) is used as a bridge or proxy to get such part-label to capsule correspondence. Then, on the right side of the figure, we show a simple network which is used to predict part labels for each capsule. The input to this one-layer architecture are the latent capsules combined with a one-hot vector of the object category. The output is the part prediction of each capsule. We use the cross entropy loss as our loss function and Adam as the optimizer with a learning rate of 0.01. Finally, the predicted part label of each capsule will be passed to its reconstructed points. Through a nearest neighbour search, we can finally classify each input point and endow it with part information.

## Experiments

The proposed 3D capsule autoencoder can learn data driven representations with novel functionalities such as part interpolation and replacement, while attaining higher performance in conventional 3D tasks such as shape classification, 3D reconstruction and part segmentation. In the sequel, we first assess the performance in object classification (§ 4.1) and follow onto 3D reconstruction and correspondence estimation (§ 4.2) without supervision where we ablate on different aspects of our architecture, before evaluating our network with task specific supervision signals such as part segmentation (§ 4.3), interpolation and replacement (§ 4.3). Note that, in each of those evaluations we use both rigid and non-rigid shape datasets.

**Table 1:** Accuracy of classification by transfer learning on the ModelNet40 dataset

	Latent-GAN (Achlioptas et al. 2018)	FoldingNet (Yang et al. 2018)	Ours-MLP-Parts	Ours-Deconv-Parts	Ours-MLP (Zhao et al. 2019)
Acc.	85.7%	88.4%	88.9%	86.8%	**89.3%**

Latent-GANAchlioptas et al. (2018), FoldingnetYang et al. (2018) and Ours-MLPZhao et al. (2019)) are trained on ShapeNet55 while the *Parts* variants are trained on the smaller ShapeNet-Parts dataset

Datasets To demonstrate the flexibility and applicability of our network we use both rigid and non-rigid shape reconstruction, segmentation and classification benchmarks. For the former (rigid case), we use the widely accepted ShapeNet-Core Chang et al. (2015), Shapenet Part Yi et al. (2016), ModelNet40 Wu et al. (2015) datasets. For the latter, we use the 3D-Coded Groueix et al. (2018b) and Dynamic Faust (Bogo et al. 2017; Yang et al. 2021) datasets for quantitative and qualitative evaluations, respectively. This is because 3D Coded provides the necessary ground truth (GT) information required for the quantitative analysis. We additionally use the human segmentation benchmark introduced by Maron et al. (2017), Poulenard and Ovsjanikov (2018) for evaluations considering body part segmentation. The specific details of these datasets as well as our evaluation schemes will be provided in the respective subsections.

In what follows, **Ours-MLP** depicts a capsule-MLP structure, while **Ours-Deconv** represents a capsule deconvolution architecture with the cluster loss activated. We also present the **Parts** variant, which corresponds to a decoder trained on ShapeNet Part dataset.

**Table 2:** Classification accuracy on ShapeNet-Part by learning on limited training data

	$1\%$	$2\%$	$5\%$	$20\%$	$100\%$
FoldingNet	56.15	67.05	75.97	84.06	88.41
Ours-mlp	59.24	67.67	76.49	84.48	89.31

The table shows the accuracies (in percentages) obtained by FoldingNet Yang et al. (2018) and our approach for different amounts of training data

### 3D Object Classification

We start by investigating the efficiency of the learned representations by evaluating the classification accuracy obtained by performing *transfer learning*. Identical to Wu et al. (2016); Achlioptas et al. (2018); Yang et al. (2018), we use a linear support vector machine (SVM) classifier in order to regress the shape class given the latent features. To do that, we reshape our latent capsules into a one dimensional feature and apply the SVM-loss. The rationale here is that each of the capsules which instantiates for a local region on the shape is informative about the object category. Combined, they form a global code. To obtain the features required for classification we pre-train our auto-encoder using the reconstruction loss. To obtain the class labels, we train our SVM on Modelnet40 Wu et al. (2015) using the same train/test split sets as Yang et al. (2018). In addition, we train our decoders on the ShapeNet Part dataset Yi et al. (2016) where the training data has 14,000 models subdivided into 16 categories. At this stage, we do not use the part information.

All the evaluation results on the classification are shown in Table 1, where our AE, trained on the smaller *Parts* dataset compared to the ShapeNet55 of Achlioptas et al. (2018); Yang et al. (2018) is capable of performing at least on par or better. This aligns with our intuition that capsules and spatial attention are useful inductive biases allowing for generalization and learning with limited data. Our capsule network can handle smaller datasets and generalize better to new tasks.

It is also noteworthy that compared to our Deconv layers, the less constrained MLP decoder Zhao et al. (2019) is more suited for such classification tasks where capturing fine-grained details are less critical. With that observation, we further evaluate the classification performance of the MLP decoder when the training data is scarce. Results presented in Table 2 indicate that even when $\sim 20\%$ of training data is used we can still attain $\sim 85\%$ accuracy and consistently outperform FoldingNet Yang et al. (2018) across different cardinalities of training split.

**Table 3:** Evaluating reconstruction quality on the non-rigid human bodies

	Oracle	PB	AtlasNet-25	AtlasNet-125	Ours-MLP	Ours-Deconv
Rigid Shape	0.85	1.91	1.56	1.51	**1.46**	**1.46**
Human	0.406	5.472	0.558	0.538	0.611	**0.436**

Oracle refers to a random sampling of the input 3D shape and constitutes an lower bound on what is achievable. The Chamfer Distance (CD) is multiplied by $10^3$ for ease of perception. PB refers to *PointNet Baseline*. AtlasNet-*K* uses *K* charts (patches) for reconstruction

### Unsupervised 3D Reconstruction

In this section, we first provide the implementation details of our auto encoder. Then we evaluate the reconstruction performance both quantitatively and qualitatively. During the qualitative evaluation, we also highlight and analysis of the spatial attention of capsules on the reconstructed point set.

Implementation Details and Datasets. Prior to training, the input point clouds are aligned to a common reference frame and size normalized. Note that, the dataset at hand is already canonicalized, so we do not apply any additional pre-processing. Chang et al. (2015) To train our AE, we use an ADAM optimizer with an initial learning rate of 0.0001 and a batch size of 8. We also employ batch normalization (BN) and RELU activation units at the point of feature extraction to generate primary capsules. Similarly, the multi-stage MLP decoder also uses BN and RELU units except for the last layer, where the activations are scaled by a $tanh(\cdot )$. During dynamic routing, we use the squash activation function as proposed in Sabour et al. (2017), Hinton et al. (2011). Note that we use the same training strategy for both MLP and Deconv decoders.

#### Quantitative Evaluations

Reconstruction Accuracy on Rigid Shapes. In a further experiment, we assess the quality of our architectures with and without cluster loss in point generation. We measure the reconstruction performance by the standard Chamfer metric and base our comparisons on the state of the art auto-encoder AtlasNet and its baselines (point-MLP) Groueix et al. (2018a). We rely on the ShapeNet Core v2 dataset Chang et al. (2015), using the same training and testing splits as well as the same evaluation metric as those in AtlasNet’s Groueix et al. (2018a). We show in Table 3 the Chamfer distances averaged over all categories and for $N>2K$ points. In the first row (*rigid shape*) of Table 3, it is observed that all of our capsule AEs result in lower reconstruction error even when a large number of patches (125) is used in favor of AtlasNet. This justifies that the proposed networks has a better summarization capability and can result in higher fidelity reconstructions.

Reconstruction Accuracy on 3D Humans. In order to evaluate the reconstruction accuracy on the non-rigid shapes, we use the synthetic Human dataset released by 3D-Coded Groueix et al. (2018b). This dataset is built upon the SMPL models with 200,000 parameters estimated in the SURREAL dataset Varol et al. (2017). There is also an added 30,000 shapes for covering edge cases such as bending over. The second row of  Table 3 makes it obvious that our proposed AE has the best performance on human reconstruction and is well applicable to both rigid and non-rigid cases. For non-rigid models, it is visible that Ours-Deconv yields better results, as handling deformations requires improved ability to capture local details.

#### Qualitative Evaluations

In the sequel, for our qualitative evaluations on the reconstruction we will be using models from the ShapeNet dataset for the rigid case and real scans from the Dynamic Faust dataset Bogo et al. (2017) for the non-rigid. Dynamic Faust Bogo et al. (2017) contains motion sequences of 10 people, each performing a set of (maximum) 14 actions. For the sake of clarity of illustration, we use a subset of Dynamic Faust with a single human subject observed in three motion sequences. In particular, we use a subset where three common motions of a single human subject are captured, namely shaking arms, jumping on one leg and running on spot. The resulting dataset contains 1102 samples and a random train-test split of [70%, 30%] is used. During training and testing, human body point clouds with 4096 points are fed to our network. Only qualitative results are presented in this experiment.

**Fig. 6:**
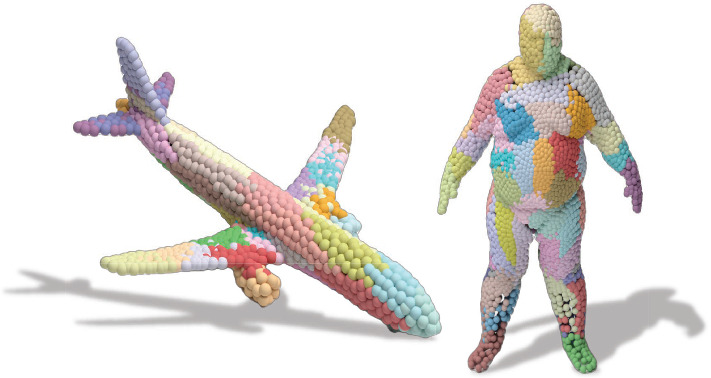
Spatial attention in 3D reconstruction. Different colored points are reconstructed from different latent capsules with our deconvolution decoder

Reconstruction with Spatial Attention. We now visualize the distribution of the points reconstructed from different capsules using the deconvolutional decoder and cluster loss. To this end, we assign a color per capsule identifying the corresponding part / region. Our results are shown in Fig. 6. It can be observed that the points reconstructed from a single capsule are spatially clustered, and that this capsule focuses its attention to reconstruct similar spatial regions. Thus we refer to this property as spatial attention of the feature representation. Note that our architecture is agnostic to the input being rigid or non-rigid.

**Fig. 7:**
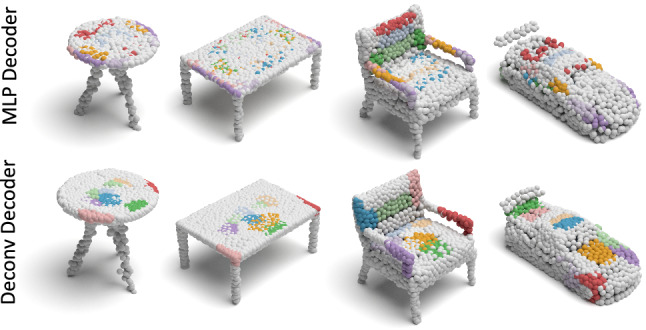
Comparison of the spatial attention in 3D reconstruction with two decoders. It is obvious that the deconvolutional decoder proposed in this paper could attain reconstructions with better clustering (local grouping) performance while the MLP-decoder suffers when part symmetries are present

**Fig. 8:**
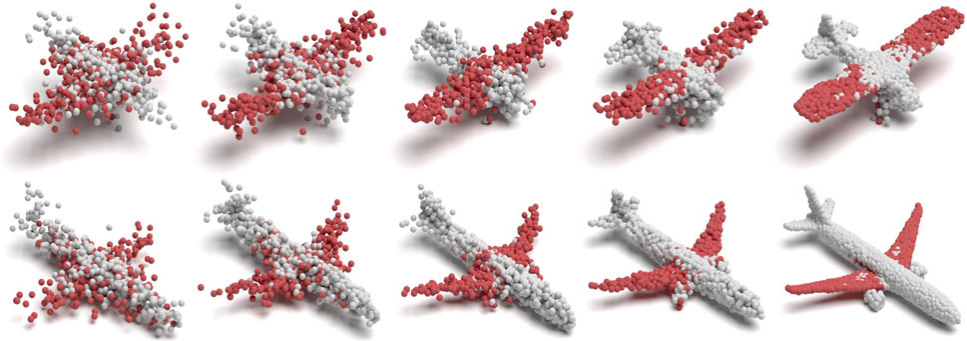
Visualizing the iterations of unsupervised AE training on the airplane object. For clear visualization, we fetch the colors belonging to the $\sim$20 capsules of the wing-part from our part predictions trained with part supervision (Color figure online)

**Fig. 9:**
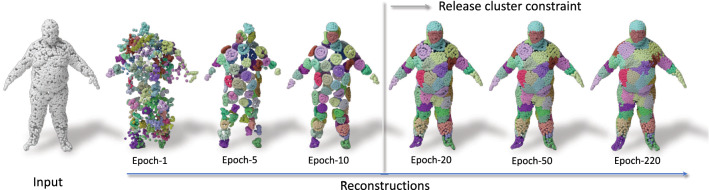
Visualizing the iterations of unsupervised AE training on the Human dataset. 64 colored points are reconstructed from corresponding 64 capsules. Before 10 epochs, the reconstruction of an capsule is clustered during training, though the cluster loss constrains the reconstruction convergence. After 10 epochs, the cluster loss is released and the reconstruction accuracy gets higher during the following training process

Effectiveness of Deconv Decoder: It is of interest to see how our deconvolutional decoder influences the spatial attention property of reconstruction. For a fair comparison to the MLP decoder, we first fix the encoder of our capsule network, and solely change the decoder architecture. For both decoders, we use the cluster loss for 10 epochs and later release it. Fig. 7 illustrates the point clustering behavior on several reconstructed shapes from the ShapeNet Part dataset, where results achieved by (i) using MLP decoder; (ii) using Deconv decoder with cluster loss, are provided. Note that, the improvement on point grouping obtained by the Deconv decoder is significant. For (i), reconstructed points distribute over the whole shape with less locality, while for (ii), points coming from each latent capsule only focus on certain regions. It is important to note that we do not use supervision for that to emerge. There are two reasons for this improvement. First, feature vectors generated by Deconv layers enjoy more continuity compared to those generated by MLP. This is also observed by Fan et al. (2017). Second, the 3D reconstruction loss (CD) has many undesirable local optima, resulting in failures of point clustering. Our cluster loss helps our model to get rid of such local optima by gathering points during initialization.

Monitoring Model Evolution: Next, we observe that, as training iterations progress, the randomly initialized capsules specialize to parts, achieving a good part segmentation at the point of convergence. We visualize this phenomenon in Fig. 8, where the capsules that have captured the wings of the airplane are monitored throughout the optimization procedure. Even though the initial random distribution is spatially spread out, the resulting configuration is still part specific. This is a natural consequence of our capsule-wise part semi supervision.

Due to its category specific nature, monitoring the evolution on human body reconstruction is more interesting and informative as we show in Fig. 9. As explained in the implementation details, during the first 10 epochs we apply our clustering loss to promote local grouping. The weight of the cluster loss then vanishes and points spread around the object. After this point, the cluster loss is released and the network aims to maximize the reconstruction accuracy. Finally, we observe the best of both worlds: the local attention is achieved and reconstructions are high-fidelity.

Patch-Wise Correspondence Estimation: As shown earlier, regardless whether we train on a single class or an entire category, the capsules succeed to specialize on parts. This motivates us to investigate if our network can be used for regional correspondence estimation.

**Fig. 10:**
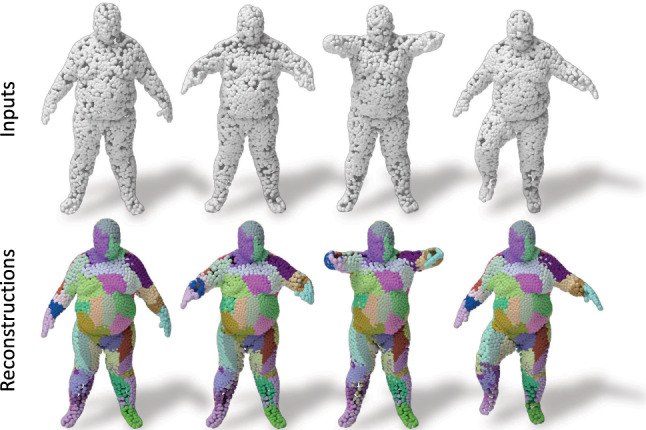
Correspondence estimation over deforming shapes. The 64 colored local patches reconstructed from 64 capsules are shown as correspondences. Our network can identify the part correspondence over a motion sequence in a fully self-supervised manner

**Fig. 11:**
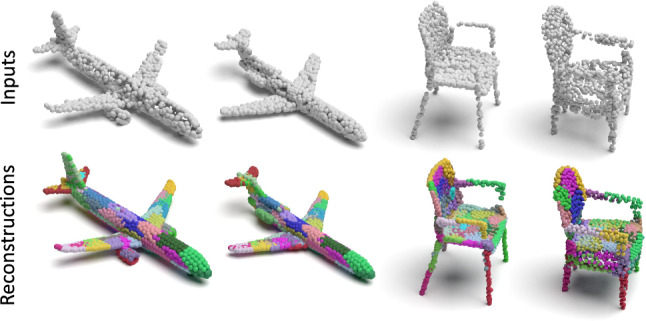
Rigid inter-class (within category) regional shape correspondence. Correspondences on 64 local patches reconstructed from 64 different capsules are visualized with color coding. Regions under correspondence are assigned identical colors. No supervision signal is utilized for correspondence estimation

First,  Fig. 10 visualizes the correspondences on the single instance case of human bodies. The local patches corresponding to each of the 64 capsules are drawn with a unique color. Hence, corresponding points should take the same color without explicit supervision. It is seen that the regions reconstructed by different capsules are consistent across different poses indicating that capsules *learn* interesting regions rather than trying to partition the ambient space. We also present a similar evaluation of patch correspondence for different instances of rigid bodies taken from the same category in Fig. 11. It can be seen that while the correct regions mostly coincide for both the airplane and the car, variations in the different instances make the fine-grained specialization hard to infer.

**Table 4:** Part segmentation on ShapeNet-Part by learning only on the $x\%$ of the training data

Metric	SONet-$1\%$	Ours-MLP-$1\%$	Ours-Deconv-$1\%$	SONet-$5\%$	Ours-MLP-$5\%$	Ours-Deconv-$5\%$
Accuracy (%)	0.78	0.85	**0.86**	0.84	0.86	**0.88**
IoU	0.64	0.67	**0.68**	0.69	**0.70**	**0.70**

**Fig. 12:**
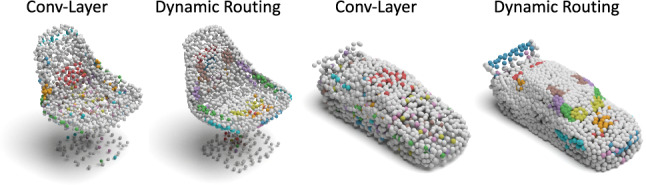
Distribution of 10 randomly selected capsules on the reconstructed shape after unsupervised autoencoder training with dynamic routing and with a simple convolutional layer

Effectiveness of Dynamic Routing: It is of interest to see whether the original argument of the capsule networks (Sabour et al. 2017; Hinton et al. 2011) claiming to better capture the intrinsic geometric properties of the object still holds in the case of our unsupervised 3D-AE. To this aim, we first show qualitative results of Ours-MLP architecture. Chair and car objects shown in Fig. 12 show that even with the lack of supervision, capsules tend to specialize on local parts of the model. While these parts may sometimes not correspond to the manually annotated part segmentation of the model, we still expect them to concentrate on semantically similar regions of the 2-manifold. Fig. 12 visualizes the distribution of 10 capsules by coloring them individually and validates our argument.

To test our second hypothesis, i.e. that a clustering arises thanks to DR, we replace the DR part of the AE with standard PointNet-like layers projecting the $1024\times 64$ PPC to 64$^2$ capsules and repeat the experiment. Fig. 12 depicts the spread of the latent vectors over the point set when such layer is employed as opposed to DR. Note that using this simple layer instead of DR both harms the reconstruction quality and yields an undesired spread of the capsules across the shape.

### Part Interpolation and Replacement

After conducting the experiment of shape part segmentation, we explore the rather uncommon but particularly interesting application of interpolating, exchanging or switching object parts via latent-space manipulation.

Thanks to the fact that 3D-PointCapsNet discovers multiple latent vectors specific to object attributes/shape parts, our network is capable of performing per-part processing in the latent space. To do that, we first spot a set of latent capsule pairs belonging to the same parts of two 3D point shapes and intersect them. Because these capsules explain the same part in multiple shapes, we assume that they are specific to the part under consideration and nothing else. Note that, as we have seen earlier, this assumption is better satisfied by the deconvolutional decoder. Acknowledging that isolating and interpolating parts in the physical space might be challenging, we then linearly interpolate between the selected capsules in the latent space. The overview of the part interpolation / replacement process is shown in  Fig. 14.

**Fig. 13:**
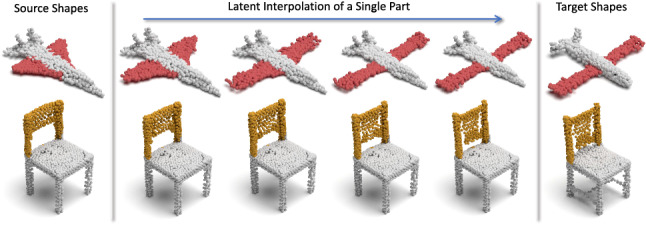
Part interpolation on the ShapeNet Part Yi et al. (2016) dataset. $\text{( }{\textbf {left}})$ The source point cloud. $\text{( }{\textbf {right}})$ Target shape. $\text{( }{\textbf {middle}})$ Part interpolation. Fixed part is marked in light blue and the interpolated part is highlighted. Capsules are capable of performing part interpolation purely via latent space arithmetic (Color figure online)

Rigid Part Editing: Shown in Fig. 13 the reconstruction of intermediate shapes vary only at a single part, the one being interpolated. It is important that the rest of the object remains unchanged even when the part is modified in the latent space. This induces promising part disentanglement ability. When the interpolator reaches the target shape it *replaces* the source part with the target one, enabling *part-exchange*. Fig. 15 further shows this in action. Given two shapes and latent capsules of the related parts, we perform a part exchange by simply switching some of the latent capsules and reconstructing them. Notably, conducting a part exchange directly on the input space by a cut-and-place would yield inconsistent shapes as the replaced parts would have no global coherence.

**Table 5:** Runtime and memory consumption of our method as well as AtlasNet

Methods	Atlas-25	Atlas-125	Ours-MLP	Ours-Deconv
Runtime (ms)	31.57	160.56	46.54	62.64
RAM (MB)	759	1573	839	841

**Fig. 14:**
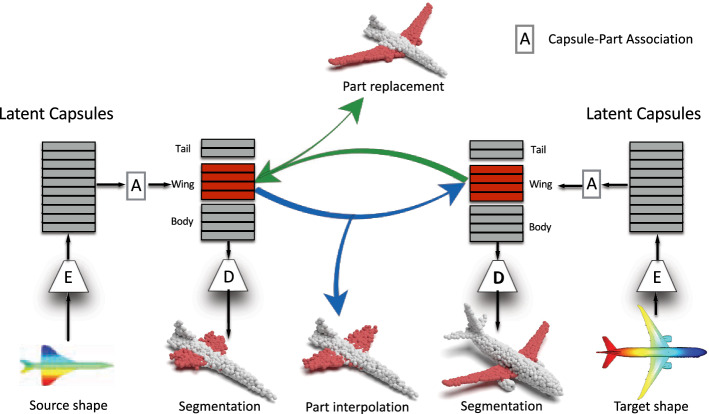
Our interpolation / replacement pipeline. Both source and target are fed to our 3D point capsule network. We then manually identify the capsules belonging to a specific part. Once the corresponding capsules are determined, they are either linearly interpolated for the purpose of part morphing or swapped for part replacement. Thanks to the fact that the entirety of the latent capsules describe the shape globally, this leads to better blending of the modified parts

**Fig. 15:**
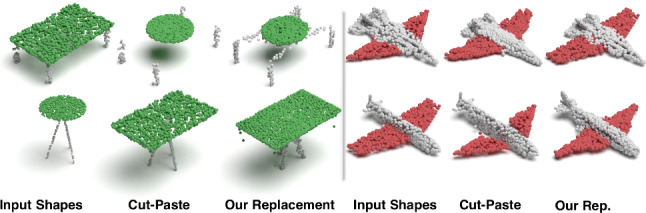
Part replacement on two rigid objects. Performing replacement in the latent space rather than Euclidean space of 3D points (cut & paste) leads to a coherent outcome

**Fig. 16:**
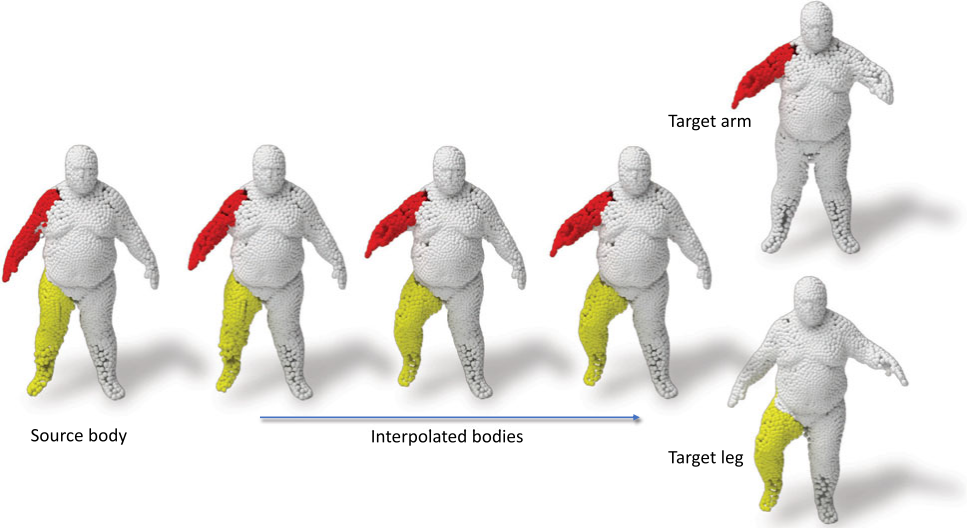
Latent interpolation in the arm & leg sub-spaces between source and target human models. Note that the unseen latent models are reconstructed accurately

Non-Rigid Part Editing: We additionally explore how blending parts of two non-rigid humans behaves within our framework. We use the identical settings and techniques as in the rigid case and interpolate the parts corresponding to a *source* and a *target* human model from the Dynamic Faust dataset Bogo et al. (2017) in the latent space of capsules. The results in  Fig. 16 show the interpolation of hands and legs between two body poses in order to generate a new pose of the same person. The red arm is interpolated form the *source body* to the *target arm* while the yellow leg is interpolated from the *source body* to the *target leg*. The generated *blended body* shows the movement or deformation from one pose to another. Note that the interpolated body is accurately reconstructed and contains rich and fine-grained details.

## Conclusion

We presented 3DPointCaps++, improving upon our previous 3D point capsule networks Zhao et al. (2019) via deconvolution decoding layers and clustering loss. Our contributions enhance the spatial attention and local focus of the capsule networks better capturing the fine grained details during reconstruction. In return, these made it possible to apply the 3D capsule networks on the perception of non-rigid 3D human bodies without any changes to the architecture or the algorithm. Our extensive evaluations also demonstrated that these new architectures excel at handling small data regimes.

Limitations & Future Work Despite all the improvements, our algorithm is limited in several aspects:

- Currently, all the capsules reconstruct the same amount of points. Moreover, a single capsule can specialize on two different parts. For these reasons, our method fails to capture very fine-grained details of the objects. As a remedy, possible future study can explore capsules with dynamically assigned number of points.
- Rotated objects pose a severe challenge for our work. To process objects with arbitrarily orientations, We can benefit from the recent rotation equivariant network design literature, e.g. *quaternion equivariant capsule networks* Zhao et al. (2020), *tensor field networks* Thomas et al. (2018) or *canonical capsules* Sun et al. (2020).
- If two shapes from the same category undergo significant topological changes, our part interpolation scheme cannot produce desirable results. Incorporating topological awareness into capsule networks is still an active field of research.
- While we explicitly consider point-representations, the literature is also rich in the sparse convolutional networks, which could be well benefit from our capsule paradigm. We leave this for future research.

Besides, in the future, we also plan to devise a routing algorithm better suited to the needs of 3D applications and will broaden our scope to scene level analysis.

## Footnotes

1 For instance, FoldingNet presents evaluations with random grids in their appendix.
